# Annexin A1 Is Increased in the Plasma of Preeclamptic Women

**DOI:** 10.1371/journal.pone.0138475

**Published:** 2015-09-23

**Authors:** Luiza O. Perucci, Fernanda S. Carneiro, Cláudia N. Ferreira, Michelle A. Sugimoto, Frederico M. Soriani, Gustavo G. Martins, Kátia M. Lima, Flávia L. Guimarães, Antônio L. Teixeira, Luci M. Dusse, Karina B. Gomes, Lirlândia P. Sousa

**Affiliations:** 1 Departamento de Análises Clínicas e Toxicológicas, Faculdade de Farmácia, Universidade Federal de Minas Gerais, Belo Horizonte, Minas Gerais, Brazil; 2 Programa de Pós-Graduação em Análises Clínicas e Toxicológicas, Universidade Federal de Minas Gerais, Belo Horizonte, Minas Gerais, Brazil; 3 Colégio Técnico, Universidade Federal de Minas Gerais, Belo Horizonte, Minas Gerais, Brazil; 4 Programa de Pós-Graduação em Ciências Farmacêuticas, Universidade Federal de Minas Gerais, Belo Horizonte, Minas Gerais, Brazil; 5 Departamento de Biologia Geral, Instituto de Ciências Biológicas, Universidade Federal de Minas Gerais, Belo Horizonte, Brazil; 6 Hospital Municipal Odilon Behrens, Belo Horizonte, Minas Gerais, Brazil; 7 Programa de Pós-Graduação em Biologia Celular, Universidade Federal de Minas Gerais, Belo Horizonte, Minas Gerais, Brazil; 8 Laboratório Interdisciplinar de Investigação Médica, Faculdade de Medicina, Universidade Federal de Minas Gerais, Belo Horizonte, Brazil; Xavier Bichat Medical School, INSERM-CNRS - Université Paris Diderot, FRANCE

## Abstract

**Background:**

Preeclampsia (PE) is a pregnancy disease associated with exacerbated inflammatory response. Annexin A1 (AnxA1) is a glucocorticoid-regulated protein endowed with anti-inflammatory and proresolving properties that has been much studied in various animal models of inflammation but poorly studied in the context of human inflammatory diseases. The main objective of this study was to measure AnxA1 levels in PE women and to compare those levels in normotensive pregnant and non-pregnant women. We evaluated the association among AnxA1, ultrasensitive C reactive protein (us-CRP) and soluble tumor necrosis factor alpha receptor type 1 (sTNF-R1) plasma levels of the study participants.

**Methods:**

This study included 40 non-pregnant, 38 normotensive pregnant and 51 PE women. PE women were stratified in early (N = 23) and late (N = 28) subgroups, according to gestational age (GA) at onset of clinical symptoms. Protein AnxA1 and us-CRP plasma levels were determined by ELISA and immunoturbidimetric assays, respectively. Transcript levels of AnxA1 in peripheral blood mononuclear cells (PBMC) were measured by real time RT-PCR.

**Results:**

Increased levels of AnxA1 coincided with higher us-CRP levels in the plasma of PE women. Pregnant women with early PE had higher levels of AnxA1 and us-CRP than normotensive pregnant women with GA <34 weeks. No significant difference was found for AnxA1 and us-CRP, comparing late PE and normotensive pregnant women with GA ≥34 weeks. AnxA1 mRNA levels in PBMC were similar among the studied groups. AnxA1 was positively correlated with sTNF-R1, but not with us-CRP.

**Conclusions:**

Our data show that increased AnxA1 levels were associated with a systemic inflammatory phenotype in PE, suggesting AnxA1 deregulation in PE pathogenesis. However, more studies are needed to clarify the role of AnxA1 and other proresolving molecules in the context of the systemic inflammatory response in this intriguing disease.

## Introduction

Preeclampsia (PE) is a multisystem disease characterized by new-onset hypertension and proteinuria on or after 20 weeks of gestation [1]. PE affects 2–8% of all pregnancies and is often associated with adverse maternal and perinatal outcomes [2]. PE has been classified according the gestational age (GA) of clinical symptoms onset in early (GA<34 weeks) or late (GA≥34 weeks) [3]. Despite years of intense research, the etiopathogenesis of PE remains to be elucidated, although placental dysfunction is considered to play a central role in the development of the disease. It has been proposed that the ischemic placenta can release soluble factors into the maternal circulation that cause endothelial cell activation and/or dysfunction and a systemic inflammatory response [4].

Redman et al. initially proposed that the features of the systemic inflammatory response observed in normotensive pregnant women are also seen in PE women, but in a greater intensity [5]. Accordingly, several studies have described increased activation of circulating leukocytes, abnormal immune cell phenotype and higher pro-inflammatory markers levels, such as C-reactive protein (CRP), in PE compared to normotensive pregnancy [6–10].

Annexin A1 (AnxA1), previously named lipocortin-1, is a 37 kDa calcium-dependent phospholipid binding protein that regulates diverse cellular functions in various cellular types [11, 12]. AnxA1 was originally described as a glucocorticoid-regulated protein with anti-phospholipase activity, but the protein exhibits many other anti-inflammatory and proresolving properties, which include inhibition of neutrophils adhesion/transmigration through the endothelium and stimulation of macrophages phagocytic clearance of apoptotic neutrophils [13]. AnxA1 is also regulated by pro-inflammatory proteins, such as lipopolysaccharide (LPS) and interleukin (IL)-6, suggesting that it may act as a brake for controlling the inflammatory response [14, 15]. Given the large body of evidence describing the anti-inflammatory and proresolving actions of AnxA1, and knowing that PE is associated with an exacerbated inflammatory state, it is plausible to hypothesize that AnxA1 may be altered in PE women.

In the present study, we evaluated AnxA1 plasma levels and AnxA1 mRNA expression in peripheral blood mononuclear cells (PBMC) in preeclamptic, normotensive pregnant and non-pregnant women. The association between AnxA1 and ultrasensitive (us)-CRP levels was tested in order to investigate the potential role of AnxA1 in the context of inflammation. In a previous study of our group, soluble tumor necrosis factor alpha (TNF-α) receptor type 1 (sTNF-R1) plasma levels were higher in PE women than in normotensive pregnant women [16]. sTNF-R1 acts as an indirect marker of TNF-α release in the circulation [17]. Therefore, we also investigated whether AnxA1 plasma levels were correlated to sTNF-R1. To date, this is the first study that evaluates AnxA1 plasma levels and mRNA expression in PBMC in PE.

## Materials and Methods

### Study participants

This case control study enrolled Brazilian women. It was approved by Ethics Committees of Universidade Federal de Minas Gerais (Institutional Review Board Project #0618.0.203.000–10) and the participant hospitals. Written informed consent was obtained from all women. Blood samples were obtained from 129 women: 40 non-pregnant, 38 normotensive pregnant and 51 preeclamptic. All pregnant women were at the third trimester of gestation.

PE was defined as blood pressure ≥ 140/90 mmHg at least in two occasions, 6 or more hours apart, and proteinuria (at least 300 mg/day or 1+ on a urine dipstick) on or after 20 weeks of gestation. Headache, oliguria, visual disturbances, upper abdominal pain, high liver enzymes and thrombocytopenia were also considered in the disease diagnosis. No cases of chronic hypertension or superimposed PE were included in this study. Preeclamptic women were stratified in two subgroups according to gestational age at clinical symptoms onset: early PE (GA<34 weeks; N = 23) and late PE (GA≥34 weeks, N = 28) [3]. The normotensive pregnant group included women with healthy pregnancies and was matched for gestational age at the time of blood sampling (GA<34 weeks: N = 14; GA≥34 weeks: N = 24). The non-pregnant group included healthy women age matched. Exclusion criteria common for all groups were: obesity (grades II and III) [18]; diabetes mellitus; cancer; coagulation disorders; cardiovascular, autoimmune, hepatic, renal and inflammatory/infectious diseases.

### Blood sampling and processing

Peripheral blood samples were collected from all women into EDTA anticoagulated tubes, which were centrifuged at 3000g for 15 minutes, room temperature. The aliquots of plasma were stored at -80°C until the analyzes for AnxA1 and us-CRP.

PBMC were isolated from the heparinized peripheral blood obtained from 12 non-pregnant, 16 normotensive pregnant and 17 PE women by Ficoll Diatrizoate gradient centrifugation (Ficoll-Paque Plus, GE Healthcare Life Sciences) at room temperature. Cells were collected, washed, resuspended in RPMI medium containing 10% of dimethyl sulfoxide and 20% of fetal bovine serum and stored at -80°C until RNA extraction.

### ELISA and immunoturbidimetric assay

AnxA1 plasma levels were determined by a commercial ELISA kit (USCN Life Sciences Inc.). An immunoturbidimetric method (Wiener Lab) was used for us-CRP plasma levels determination. The assays were performed according to manufacturers’ instructions. Samples from PE, normotensive pregnant and non-pregnant groups were run simultaneously and the assays were blinded for patient identification and disease status. The values are represented as AnxA1 μg/mL and us-CRP mg/L of plasma

### Comparative real-time PCR

RNA was extracted from isolated PBMC using a commercial kit (RNeasy Mini Kit, Qiagen). An aliquot of RNA (130ng) was reverse transcribed into cDNA using reverse transcriptase (Superscript III, Invitrogen), following the manufacturers’ protocols. Real-time PCR assay was carried out in duplicate in a 10μL volume using SYBR Green (Applied Biosystems) and the following primers: human annexin A1 (AnxA1) forward 5’-ATCAGCGGTGAGCCCCTATC-3’ and reverse 5’-TTCATCCAGGGGCTTTCCTG-3’ and human glyceraldehyde-3-phosphate dehydrogenase (GAPDH) forward 5’-GGTCGGAGTCAACGGATTTG-3’ and reverse 5’-ATGAGCCCCAGCCTTCTCCAT-3’. Relative AnxA1 mRNA expression levels were calculated from normalized ΔCT (cycle threshold) related to the housekeeping gene (GAPDH). For the detection of changes in gene expression in non-pregnant and PE groups the normalized ΔCT values for each sample were compared with the mean ΔCT level of the normotensive pregnant group and the change expression of AnxA1 gene (ΔΔCT) was calculated. The normotensive pregnant group was considered as the reference group because the main purpose of this analysis was to evaluate AnxA1 transcript levels in PE women in comparison to normotensive pregnant women. The obtained values were converted to a linear scale (2^-ΔΔCT^) and reported as the fold-change in expression (arbitrary units).

### Statistical analysis

Statistical analysis was performed using SPSS 19.0 for Windows (Chicago, IL, USA). The normality of continuous variables was assessed using Shapiro-Wilk’s W-test. Continuous variables did not follow a normal distribution and were analyzed by nonparametric Kruskal-Wallis test or Mann-Whitney U-test with Dunn-Bonferroni’s post hoc correction. Comparison of categorical variables was performed by Pearson chi-square (X^2^) test. Correlation analysis was evaluated by Spearman coefficients (Rs) and included all the participants of the study. All statistical analyzes were performed using a significance level of α = 0.05.

## Results

### Clinical characteristics

Clinical characteristics of the studied participants are described in Table 1. There were no significant differences comparing age and body mass index among the three studied groups. PE women had higher gestational weight gain and increased percentage of primiparas compared to normotensive pregnant women. As expected, both systolic and diastolic blood pressures were significantly increased in PE than in the normotensive pregnant and non-pregnant women.

**Table 1:** Clinical characteristics of the studied participants.

Variables	NP (N = 40)	Norm (N = 38)	PE (N = 51)	P value
**Age (years)** ^a^	25 (22–30)	27 (21–30)	26 (21–30)	0.970^1^
**BMI (Kg/m** ^**2**^ **)** ^a^	21.4 (20.1–24.9)	22.8 (20.3–26.1)	23.5 (20.5–25.2)	0.157^1^
**GWG (Kg)** ^a^	N/A	10.4 (7.4–13.5)	13.0 (9.6–20.9)	0.005^1^ *
**GA at blood draw (weeks)** ^a^	N/A	36 (31–39)	34 (31–37)	0.353^1^
**Primiparas (%)** ^b^	N/A	12 (32)	27 (53)	0.045^2^ *
**SBP (mmHg)** ^a^	120 (110–120)	110 (100–110)	160 (160–170)	< 0.001^1^ *
**DBP (mmHg)** ^a^	80 (70–80)	70 (70–78)	110 (100–110)	<0.001^1^ *

BMI (body mass index: before pregnancy), GWG (gestational weight gain), GA (gestational age), SBP (systolic blood pressure), DBP (diastolic blood pressure), NP (non-pregnant women), Norm (normotensive pregnant women), PE (preeclamptic women), N/A (not applicable).

^a^Data are presented as median (25th–75th percentiles).

^b^Data are presented as number (percentage).

^1^Kruskal-Wallis/Mann-Whitney test with Dunn-Bonferroni’s correction

^2^Pearson chi-square (X^2^) test.

*p<0.05.

### AnxA1 protein and mRNA levels

**Fig 1:**
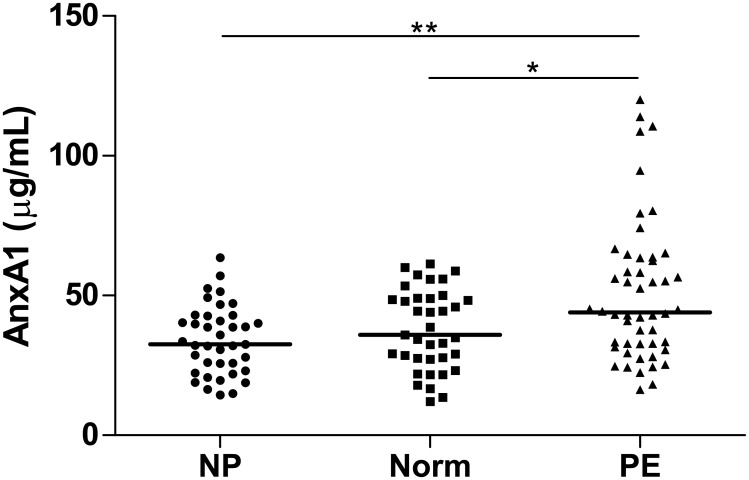
AnxA1 plasma levels in non-pregnant, normotensive pregnant and PE women. NP (non-pregnant women), Norm (normotensive pregnant women), PE (preeclamptic women). Horizontal bars represent median values for AnxA1 (micrograms/milliliter). **P<0.01, *P<0.05. Plasma levels of AnxA1 were higher in PE women than in normotensive pregnant and non-pregnant women. No significant differences were found comparing non-pregnant and normotensive pregnant women.

AnxA1 plasma levels were higher in PE women [median (25th–75th percentiles), 43.2 (30.8–57.8)μg/mL] comparing to non-pregnant women [25.9 (18.9–32.5)μg/mL] (P = 0.001). PE women also showed higher levels of AnxA1 compared to normotensive pregnant women [30.1 (19.0–35.7)μg/mL] (P = 0.026). No difference was found in AnxA1 levels when comparing non-pregnant and normotensive pregnant groups (Fig 1).

**Fig 2:**
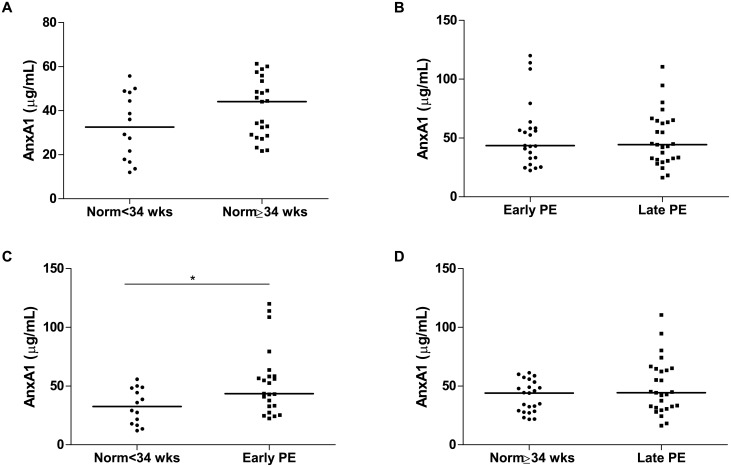
AnxA1 plasma levels in normotensive pregnant and PE women according to gestational age. Norm<34 wks (normotensive pregnant women with GA<34 weeks), Norm≥34 wks (normotensive pregnant women with GA≥34 weeks), PE (preeclampsia). Horizontal bars represent median for AnxA1 levels (micrograms/milliliter). *P<0.05. AnxA1 plasma levels were higher in pregnant women with early PE than in normotensive pregnant women with GA<34 weeks (C). No significant differences were detected between the normotensive pregnant subgroups (A), early and late PE (B) and normotensive pregnant women with GA≥34 weeks and pregnant women with late PE (D).

AnxA1 plasma levels were similar between normotensive pregnant women (GA<34 weeks) [32.6 (17.6–48.5)μg/mL] and normotensive pregnant women (GA≥34 weeks) [44.1 (28.6–53.4)μg/mL] (Fig 2A). No significant difference was found in AnxA1 plasma levels between early PE [43.5 (32.8–58.5)μg/mL] and late PE [44.3 (31.6–64.7)μg/mL] (Fig 2B). Pregnant women with early PE had higher AnxA1 levels compared to normotensive pregnant women (GA<34 weeks) (P = 0.020) (Fig 2C). However, AnxA1 levels were no significantly different between pregnant women with late PE and normotensive pregnant women (GA≥34 weeks) (Fig 2D).

**Fig 3:**
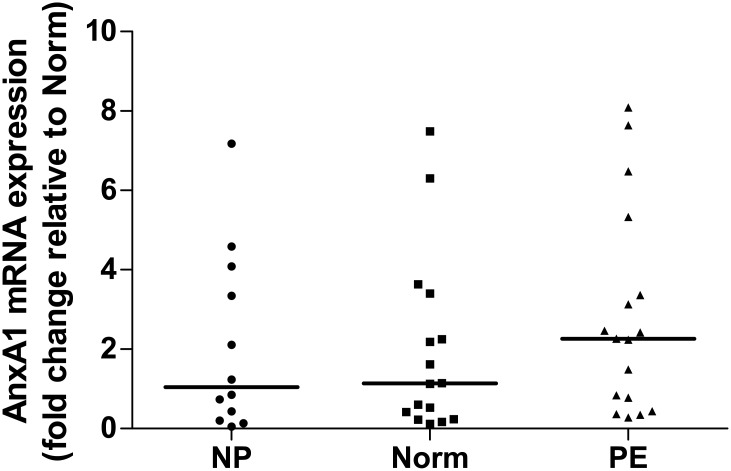
AnxA1 mRNA expression in PBMC of non-pregnant, normotensive pregnant and PE women. NP (non-pregnant women), Norm (normotensive pregnant women), PE (preeclamptic women). Horizontal bars represent median values for Anx1 mRNA fold change expression relative to the calibrator group (Norm). The transcript levels of AnxA1 were similar among the study groups.

Measurement of AnxA1 gene expression in PBMC from studied groups showed that AnxA1 mRNA levels were similar among them (Fig 3).

### Us-CRP

**Fig 4:**
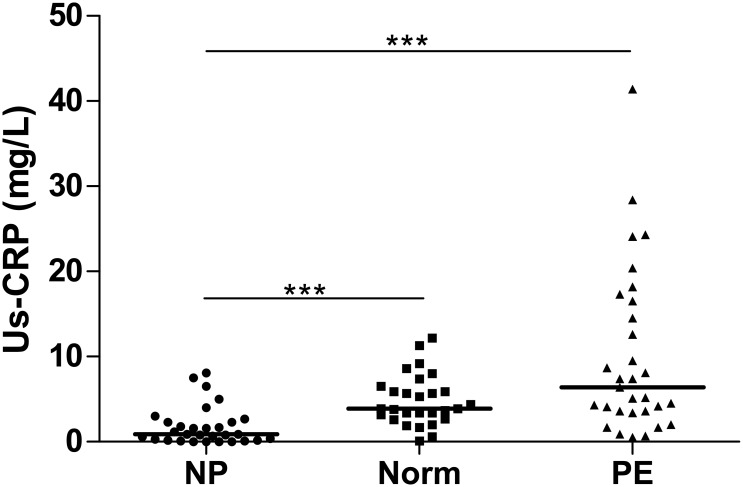
us-CRP plasma levels in non-pregnant, normotensive pregnant and PE women. NP (non-pregnant women), Norm (normotensive pregnant women), PE (preeclamptic women). Horizontal bars represent median values for us-CRP (milligrams/liter). ***P<0.001. Plasma levels of us-CRP were higher in normotensive pregnant women compared to non-pregnant women and in PE women than in non-pregnant women. No significant difference was detected between PE and normotensive pregnant women.

**Fig 5:**
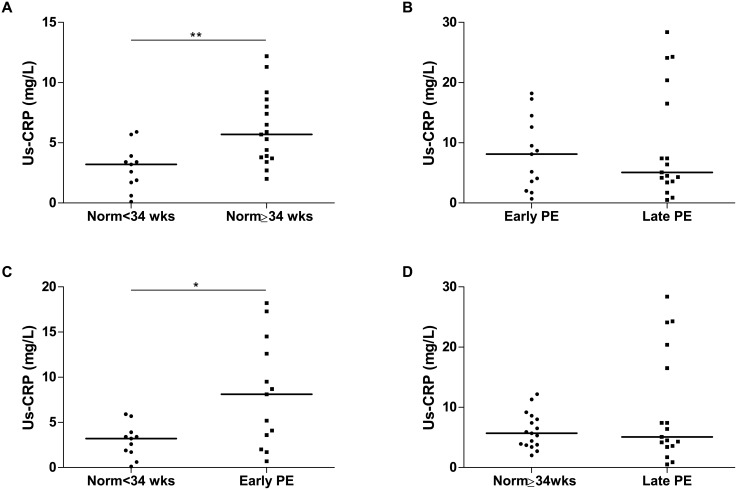
us-CRP plasma levels in normotensive pregnant and PE women according to gestational age. Norm<34 wks (normotensive pregnant women with GA<34 weeks), Norm≥34 wks (normotensive pregnant women with GA≥34 weeks), PE (preeclamptic women). Horizontal bars represent median us-CRP levels (milligrams/liter). *P<0.05. Plasma levels of us-CRP were higher in normotensive pregnant women (GA≥34 weeks) than in normotensive pregnant women (GA<34 weeks) (A). Pregnant women with early PE had higher levels of us-CRP than normotensive pregnant women with GA<34 weeks (C). No significant differences were found between pregnant women with early and late PE (B) and between pregnant women with late PE and normotensive pregnant women GA≥34 weeks (D).

Us-CRP plasma levels were higher in PE women [5.8 (3.6–15.0)mg/L] than in non-pregnant women [0.9 (0.2–2.4)mg/L] (P<0.001) and in normotensive pregnant women [3.9 (2.8–6.4)mg/L] compared to non-pregnant women (P<0.001). No significant difference was found in us-CRP levels between PE women and in normotensive pregnant women, without stratifying the groups according to gestational age (Fig 4).

us-CRP levels were higher in normotensive pregnant women (GA≥34 weeks) [5.7 (3.8–8.3)mg/L] comparing to normotensive pregnant women (GA<34 weeks) [3.2 (1.7–3.9)mg/L] (P = 0.002) (Fig 5A). Us-CRP levels were not significantly different between early PE [8.1 (2.8–13.6)mg/L] and late PE [5.1 (3.5–18.5)mg/L] (Fig 5B). Pregnant women with early PE had higher us-CRP levels compared to normotensive pregnant women (GA<34 weeks) (P = 0.018) (Fig 5C). However, us-CRP levels were similar between late PE and normotensive pregnant (GA≥34 weeks) (Fig 5D).

### Correlations among AnxA1, us-CRP and sTNF-R1 plasma levels

In a prior study, we showed that sTNF-R1 plasma levels were higher in PE women [3479 (3182–4339) pg/mL) than in normotensive pregnant women [3028 (2468–3606) pg/mL] (P = 0.014) [16]. In this study we performed correlations among sTNF-R1, AnxA1 and us-CRP levels and found a positive correlation between AnxA1 and sTNF-R1 (R = 0.352, P = 0.002), and between us-CRP and sTNF-R1 (R = 0.555, P<0.001). On the other hand, AnxA1 and us-CRP plasma levels did not correlate.

## Discussion

Our data showed that AnxA1, an anti-inflammatory and proresolving protein, is increased in preeclamptic women.

It is know that normotensive pregnancy is characterized by a state of mild systemic inflammation [5]. Consistent with this observation, we have found increased levels of us-CRP, a marker of systemic inflammatory response, in normotensive pregnancy compared to the non-pregnant state. Moreover, systemic inflammatory response strengthens as pregnancy advances, peaking during the third trimester of gestation [19]. Accordingly, in our study normotensive pregnant women (GA≥34 weeks) had higher us-CRP levels than normotensive pregnant women (GA<34 weeks).

PE has been associated to a greater inflammatory response [5]. In fact, us-CRP levels were increased in early PE than in normotensive pregnancy (GA<34 weeks). This result was not observed for the late PE subgroup. Strong evidence suggests that early PE is clinically a more severe disease than late PE [20, 21], consistent with our finding that us-CRP levels were different only when comparing early PE and the respective control.

It is well established that there is a physiological balance between pro-inflammatory and regulatory responses in normotensive pregnancies [9], which means that regulatory molecular pathways are sufficient to attenuate the mild inflammatory response. In PE, it has been proposed that this balance shifts toward a pro-inflammatory state and there is a failure to regulate the inflammation [5, 9]. AnxA1 is a protein that limits initial steps of inflammation and also acts on the resolution phase of the inflammatory response [13, 22, 23]. Our data may imply that Anx1 is increased in PE women in an attempt to attenuate the exacerbated inflammatory response in these patients. Although AnxA1 and us-CRP did not correlate to each other, both factors were elevated in PE women. In addition, AnxA1 and sTNF-R1 (an inflammatory marker) levels were positively correlated. AnxA1 levels in normotensive pregnant women were comparable to those levels in non-pregnant women. Considering that inflammation is physiological in normotensive pregnancies, it is plausible to suggest that proresolving mechanisms are not increased during physiological conditions, but they arise when the inflammatory status increase, as in the case of pathological conditions such as PE. Other studies have shown increased levels of pro-inflammatory markers, such as tumor necrosis factor alpha (TNF-α), interleukin (IL)-6 and IL-1β, in early PE than in late PE and normotensive pregnancy [24,25]. Our findings corroborate the possible regulatory role of AnxA1 mainly in the severe clinical forms of the disease (i.e., early PE), in which the inflammatory response is exacerbated.

Chronic inflammation in PE suggests that the resolution of inflammation is dysfunctional. Accordingly, increased AnxA1 levels seem to be insufficient to resolve inflammation. Indeed, systemic levels of proresolving mediators are increased in others chronic inflammatory diseases such as inflammatory bowel disease and Alzheimer’s disease [26, 27], suggesting that this resolution pathway is dysfunctional. Proresolving and anti-inflammatory actions of AnxA1 are mediated by a G-protein-couple receptor named formyl peptide receptor like-2 (FPR2)/lipoxin A4 receptor (ALXR), hereafter referred as “ALX” [28]. Cooray et al. [29] showed that ALX/ALX dimer signature is activated by AnxA1 trough p38/MAPKAPK/Hsp27/IL-10 pathway. These authors argue that AnxA1 up-regulation may be ineffective to resolve inflammation if ALX/ALX dimerization fails. Additionally, lower ALX expression level might be associated with reduced anti-inflammatory and proresolutive responses to AnxA1. Decreased ALX expression has been observed in patients with asthma, a chronic inflammatory disease [30]. Simieli et al. described a single nucleotide mutation (A/G) in the core promoter of ALX gene that reduced ~35–90% of its activity *in vitro*. ALX promoter activity may also be repressed by methylation [31]. These mechanisms might explain the apparent ineffectiveness of AnxA1 up-regulation in some human inflammatory/vascular diseases [32–34]. It remains to be investigated whether these dysfunctional mechanisms in AnxA1 resolution pathway are present in PE.

We also investigated AnxA1 gene expression in PBMCs in order to evaluate one possible source of the protein in the circulation. No significant difference was detected among the studied groups, with a tendency of higher expression of AnxA1 mRNA in PE women. AnxA1 is found predominantly within differentiated cells, like neutrophils, monocytes/macrophages and mast cells, but the exact source of plasma AnxA1 has not been determined yet [35]. This protein is expressed in the placenta, predominantly in the synciciotrophoblast [36], therefore it may be another source of AnxA1 in the plasma. To date, no study has evaluated the differential expression of AnxA1 in the placenta of normotensive and preeclamptic women, a matter under investigation in our lab.

To the best of our knowledge this is the first work that shows higher AnxA1 levels in preeclamptic women. Moreover, patients with PE were stratified according to gestational age at clinical symptoms onset, which allowed evaluating AnxA1 in different mechanisms of disease. One limitation of this study is that AnxA1 transcript levels were not evaluated in all women and pregnant groups were not stratified according to the gestational age in this analysis due to the small sample size. Moreover, AnxA1 and us-CRP levels may be influenced by ethnicity and smoking. These parameters were not evaluated in our study due to the high genetic variability present in Brazilian population, and because it was difficult to obtain accurate information about women’s smoking status. Unfortunately, we were unable to measure the exposure to cigarette smoking in this study using methods that evaluate nicotine metabolites, such as an ELISA assay for Cotinine, because a new collection of samples would be required for this purpose, which would preclude the realization of correlation analyzes with AnxA1 and us-CRP levels for each participant of the study. The dosage of Cotinine, as well as other pro-resolving molecules, such as Lipoxin A4, will be a matter of future studies in our group. Finally, patients taking medications that could potentially impact the analyzes were not excluded in this study, since polytherapy is common in patients with PE.

Concluding, it is possible that AnxA1 participate in PE pathogenesis, but more studies are needed to clarify the role of AnxA1 and other proresolving molecules in the context of the systemic inflammatory response in this intriguing disease. The potential use of AnxA1 as a biomarker in PE should be better investigated in prospective studies.

## Supporting Information

S1 File Raw data from measurements of AnxA1 (protein and mRNA levels), us-CRP levels, sTNF-R1 levels, as well as clinical parameters (age, gestational age, number of pregnancies, body mass index, gestational weight gain, systolic and diastolic blood pressure) of the participants in this study. (XLS)
